# MicroRNA-770-5p contributes to podocyte injury via targeting E2F3 in diabetic nephropathy

**DOI:** 10.1590/1414-431X20209360

**Published:** 2020-07-17

**Authors:** Juanjuan Guo, Jie Han, Jieying Liu, Shaoli Wang

**Affiliations:** 1 Department of Geriatric Ward, Heping Hospital Affiliated to Changzhi Medical College, Changzhi, Shanxi, China; 2 Department of Physical Examination Center, Heping Hospital Affiliated to Changzhi Medical College, Changzhi, Shanxi, China

**Keywords:** miR-770-5p, E2F3, Diabetic nephropathy, Podocyte, Proliferation, Apoptosis

## Abstract

Diabetic nephropathy (DN) has been identified as the major cause of end-stage renal disease (ESRD) in most developed countries. MicroRNA-770-5p depletion could repress high glucose (HG)-triggered apoptosis in podocytes, and downregulation of E2F transcription factor 3 (E2F3) could facilitate podocyte injury. Nevertheless, whether E2F3 is involved in miR-770-5p knockdown-mediated improvement of DN is still unclear. The expression levels of miR-770-5p and E2F3 were detected in HG-treated podocytes by RT-qPCR. The expression levels of E2F3, apoptosis-related proteins Bcl-2 related X protein (Bax), B-cell lymphoma-2 (Bcl-2), Bad, apoptotic peptidase activating factor 1 (APAF1), C-caspase3, C-caspase7, and C-caspase9 were detected by western blot assay. The effects of miR-770-5p and E2F3 on HG-treated podocytes proliferation and apoptosis were detected by CCK-8 and flow cytometry assays. The interaction between miR-770-5p and E2F3 was predicted by Targetscan, and then verified by the dual-luciferase reporter assay. MiR-770-5p was upregulated and E2F3 was downregulated in HG-treated podocytes. MiR-770-5p inhibited proliferation and promoted apoptosis and E2F3 promoted proliferation and suppressed apoptosis in HG-treated podocytes. E2F3 is a target gene of miR-770-5p and it partially abolished the effect of miR-770-5p in HG-triggered proliferation and apoptosis of podocytes. MiR-770-5p deficiency blocked HG-induced APAF1/caspase9 pathway via targeting E2F3 in podocytes. We firstly confirmed that E2F3 was a target of miR-770-5p in podocytes. These findings suggested that miR-770-5p expedited podocyte injury by targeting E2F3, and the miR-770-5p/E2F3 axis might represent a pathological mechanism of DN progression.

## Introduction

Diabetic nephropathy (DN) is a type of microvascular complication in diabetes, and has been identified as the major cause of end-stage renal disease (ESRD) in most developed countries (1). Approximately 40% of patients with diabetes develop DN, and the diabetes-related deaths in adults were more than 0.39 million only in 2010 (1,2). With a rapidly increasing incidence, diabetes has become an important public health concern (3 [Bibr B04] [Bibr B05]). Podocytes, a class of highly differentiated glomerular epithelial cell, exert the vital function of maintaining the integrity of the glomerular filtration barrier (4,5). Previous studies showed that the injury and loss of podocytes is strictly related to the early pathological mechanism of DN (6). Despite the substantial progress in therapeutic methods, it remains imperative to further explore podocyte-based therapies that can prevent or cure DN.

MicroRNAs (miRNAs or miRs) are a class of short non-coding, highly conserved RNAs with 19-25 nucleotides in length, which can bind to the 3′-untranslated region (3′UTR) of target mRNAs, and thereby promote mRNA degradation or mRNA translation inhibition (7). Evidence indicates that miRNAs play crucial roles in various types of biological and cellular processes, including proliferation, apoptosis, inflammation, and signal transduction (8–10). In recent years, miRNAs have been confirmed to be implicated in pathologies and are gradually becoming a new-generation therapeutic target for DN (11,12). For example, overexpression of miR-181b contributed to cell survival and suppressed apoptosis by binding to TIMP3 in DN (13), and miR-204-3p had a protective effect by hindering high glucose (HG)-induced apoptosis and dysfunction in podocytes (14). MiR-770-5p, a form of mature miR-770, has been reported to be highly expressed in HG-treated podocytes. In addition, depletion of miR-770-5p could repress HG-triggered apoptosis by binding to TRIAP1 in podocytes (15), suggesting that miR-770-5p might act as a damaging factor in DN.

E2F transcription factor 3 (E2F3), a member of the E2F family, has been pointed out to be tightly linked to proliferation and apoptosis (16,17). Furthermore, a previous study reported that E2F3 was down-regulated in HG-treated podocytes, and downregulation of E2F3 could facilitate podocyte injury, indicating that E2F3 might serve as a protective factor in DN (18). Nevertheless, whether E2F3 is involved in miR-770-5p knockdown-mediated improvement on podocytes is still unclear.

In this study, our data showed that miR-770-5p repressed proliferation and promoted apoptosis of HG-treated podocytes. Moreover, through bioinformatics analysis, we first discovered that miR-770-5p possessed some complementary sites with E2F3-3′UTR in podocytes. Hence, we aimed to assess whether miR-770-5p could regulate HG-treated podocyte injury by targeting E2F3.

## Material and Methods

### Podocyte culture

Human podocytes were purchased from Otwo Biotech (China), and were maintained in McCoy's 5A medium (Invitrogen, USA) containing 10% fetal bovine serum (FBS, Invitrogen) and 1% antibiotic (100 U/mL penicillin and 100 μg/mL streptomycin, Solarbio, China) in an incubator containing 5% CO_2_ at 37°C. Additionally, podocytes were first cultured in the medium without serum for 12 h, then treated podocytes were exposed for 0, 24, and 48 h with 30 mM D-glucose (high glucose, HG), 5 mM D-glucose (normal glucose, NG), or 30 mM mannitol (control group), based on a previous paper that had used 33 mM for HG and mannitol (19). The purity of the medium was detected by a clinical diagnostic PCR kit (TaKaRa, Japan) according to a previous description (20 [Bibr B21]).

### Cell transfection

MiR-770-5p mimic (miR-770-5p), miR-770-5p inhibitor (anti-miR-770-5p), siRNA against E2F3 (si-E2F3), and negative controls (miR-NC, anti-miR-NC, si-NC) were obtained from GenePharma (China). For overexpression of E2F3, the cDNA sequence of E2F3 was inserted into pcDNA3.1 empty vector (Invitrogen), termed as pcDNA3.1-E2F3 (E2F3). All oligonucleotides and plasmids were transfected into podocytes (2×10^5^ cells/well) with Lipofectamine 2000 reagents (Invitrogen), according to the operation manual.

### RNA extraction and real-time quantitative PCR (RT-qPCR)

Total RNA was isolated from podocytes (3×10^6^ cells/well) in accordance with the instructions for TRIzol reagent (Invitrogen). Extracted RNA samples (miR-770-5p and E2F3) were reversed into complementary DNA (cDNA) using M-MLV reverse transcriptase (Promega, USA). The cDNA amplification of miR-770-5p and E2F3 was implemented with SYBR^®^ PremixExTaq^TM^ reagent (TaKaRa) on ABI Prism^®^ 7500 Sequence Detection System (Applied Biosystems, USA). The relative expression levels of miR-770-5p and E2F3 were calculated by the 2^-ΔΔCt^ method. U6 small nuclear RNA (snRNA) and glyceraldehyde-3-phosphate dehydrogenase (GAPDH) were used as internal references to normalize miR-770-5p orE2F3 expression, respectively. The following primer sequences were used in RT-qPCR:miR-770-5p (ABI miRNA specific primers, ABI#002002); E2F3: 5′-TATCCCTAAACCCGCTTCC-3′ (sense), 5′-TTCACAAACGGTCCTTCTA-3′ (antisense); U6: 5′-GCTTCGGCAGCACATATACTAAAAT-3′ (sense), 5′-CGCTTCACGAATTTGCGTGTCAT-3′ (antisense); GAPDH: 5′-AGAAGGCTGGGGCTCATTTG-3′ (sense), 5′-AGGGGCCATCCACAGTCTTC-3′ (antisense).

### Cell proliferation assay

Podocyte viability was detected using cell counting kit-8 assay (CCK-8, Beyotime Institute of Biotechnology, China) according to the operation manual. Briefly, 8×10^3^ transfected podocytes were seeded in 96-well plates and cultured for 24 h at 37°C. Then, 10 mL CCK-8 solution was added to each well, followed by incubation for another 4 h. The absorbance values at 450 nm were then detected under a microplate reader.

### Cell apoptosis assay

The flow cytometry assay was carried out to detect the effects of HG, miR-770-5p, and E2F3 on the apoptosis rate of podocytes. After 48 h of transfection, podocytes (5×10^5^ cells/well) were harvested and washed twice with cold PBS (Invitrogen), and resuspended with 100 μL binding buffer. After treatment, podocytes were stained with 5 μL annexin (V-fluorescein isothiocyanate) and propidium iodide (PI) (Thermo Fisher Scientific, USA), and incubated in the dark for 15 min at room temperature. Finally, the stained podocytes were detected with FACScan flow cytometry (BD Bioscience, USA) according to the operation manual.

### Western blot assay

Protein expression levels of E2F3, Bcl-2 related X protein (Bax), B-cell lymphoma-2 (Bcl-2), Bad, apoptotic peptidase activating factor 1 (APAF1), C-caspase3, C-caspase7, and C-caspase9 were measured using western blot assay in podocytes under different treatments. Transfected podocytes (2×10^6^ cells/well) were lysed using pre-cold RIPA buffer (Beyotime Institute of Biotechnology) according to the manufacturer instructions. Podocyte lysates were separated in 10% sodium dodecyl sulfate-polyacrylamide gel electrophoresis (SDS-PAGE), and then isolated proteins were transferred onto a polyvinylidene fluoride (PVDF) membrane (Millipore, USA). After blocking the proteins with 5% non-fat milk in the membranes for 1 h at room temperature, membranes were incubated with primary antibodies against E2F3 (ab152126, 1:2000 dilution), Bad (ab32445, 1:1000 dilution), Bcl-2 (ab32124, 1:1000 dilution), Bax (ab69643, 1:1000 dilution), APAF1 (ab2001, 1:5000 dilution), C-caspase3 (ab4051, 1:1000 dilution), C-caspase7 (ab32522, 1:1000 dilution), C-caspase9 (ab2014, 1:1000 dilution), and GAPDH (1:1000, ab9485) (all from Abcam, UK) overnight at 4°C. The horseradish peroxidase (HRP)-linked secondary antibody (ab205718, 1:10,000 dilution, Abcam) was then incubated with the membranes at room temperature for 1 h. Finally, an ECL detection kit (Pierce Biotechnolgy, USA) was used to detect the protein bands.

### Dual-luciferase reporter assay

Partial E2F3 3′UTR fragment containing putative miR-770-5p targeting site was obtained and cloned into pmirGLO vector (Promega), termed as WT-E2F3-3′UTR (wild type) and MUT-E2F3-3′UTR (mutant) reporter plasmids. Subsequently, podocytes (1×10^5^ cells/well) were co-transfected with WT-E2F3 or MUT-E2F3 and miR-NC or miR-770-5p using Lipofectamine 2000 reagents (Invitrogen) followed by incubation at 37°C for 48 h. Lastly, luciferase activities in podocyte lysates were analyzed with the dual-luciferase reporter assay kit (Promega).

### Statistical analysis

Statistical analysis was carried out with SPSS 20.0 software (IBM, USA). Each independent experiment was repeated three times. Statistical comparisons were analyzed using Student's *t*-test or one way analysis of variance (ANOVA). Data are reported as means±SD. Statistical significance was considered when P<0.05.

## Results

### miR-770-5p expression was upregulated and E2F3 expression was downregulated in HG-treated podocytes

**Figure 1:**
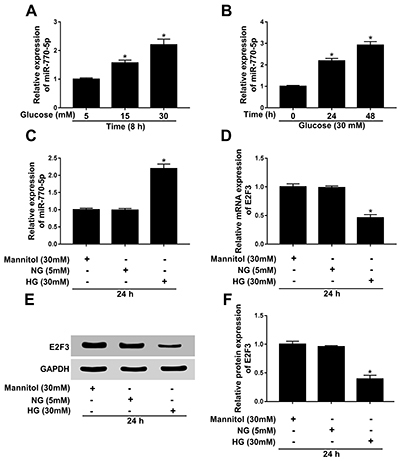
Expression of miR-770-5p and E2F3 was tested in high glucose (HG, 30 mM)-treated podocytes. **A**, The expression level of miR-770-5p was detected by RT-qPCR in podocytes treated with 5, 15, and 30 mM glucose for 8 h. **B**, miR-770-5p expression level was measured by RT-qPCR in podocytes treated with 30 mM glucose for 0, 24, and 48 h. **C** and **D**, miR-770-5p and E2F3 expression levels were assessed by RT-qPCR in podocytes treated with normal glucose (NG, 5 mM) or HG for 24 h. **E** and **F**, E2F3 protein level was detected by western blot in podocytes treated with NG or HG for 24 h. Data are reported as means±SD. *P<0.05 (ANOVA).

To investigate the impact of HG on miR-770-5p and E2F3 in podocytes, podocytes were treated under multiple conditions. Hyperglycemia is considered to be the main initiating factor in irreversible kidney injury. Hence, *in vitro*, we selected HG-treated podocytes as the model for DN study (20). Firstly, podocytes were treated with various doses of glucose. miR-770-5p expression was highly expressed in podocytes treated with 30 mM glucose relative to cells treated with 5 and 15 mM glucose (Figure 1A). Then, 30 mM glucose-treated podocytes were cultured in a time-dependent method. miR-770-5p was markedly upregulated at 24 and 48 h compared to 0 h (Figure 1B). Therefore, 30 mM glucose (termed HG) treatment for 24 h was chosen to probe the following experiments in podocytes. As shown in Figure 1C and D, miR-770-5p was highly expressed and E2F3 expression was downregulated in HG-treated podocytes for 24 h, compared to treatment with 5 mM glucose (normal glucose (NG)). Moreover, we further confirmed that the protein level of E2F3 was downregulated in HG-treated podocytes versus the control group (Figure 1E and F). These data suggested that HG induced miR-770-5p and blocked E2F3 in podocytes.

### miR-770-5p inhibited proliferation and promoted apoptosis of HG-treated podocytes *in vitro*

**Figure 2:**
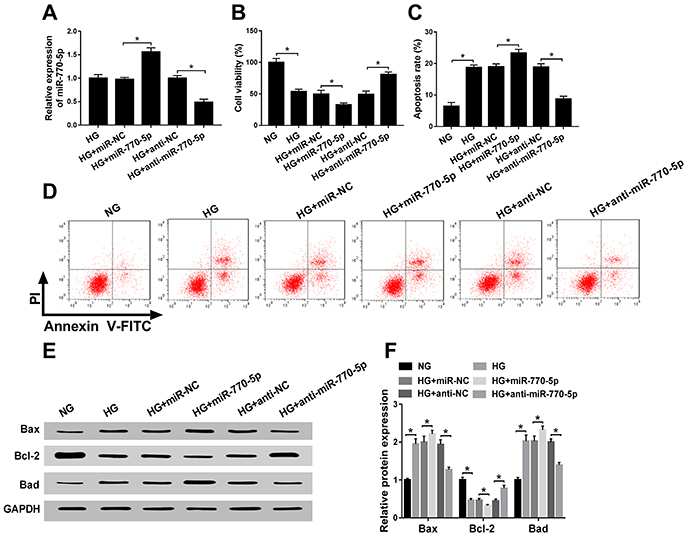
miR-770-5p suppressed proliferation and facilitated apoptosis of high glucose (HG)-treated podocytes *in vitro*. **A**, Transfection efficiency of miR-770-5p or anti-miR-770-5p in HG-treated podocytes. **B**, Cell viability was detected by CCK-8 assay in treated podocytes. **C** and **D**, Apoptosis rate was assessed by flow cytometry in treated podocytes. **E** and **F**, The levels of Bax, Bcl-2, and Bad were determined by western blot assay in treated podocytes. Data are reported as means±SD. *P<0.05 (ANOVA). NG: normal glucose (5 mM); HG: high glucose (30 mM); NC: negative control.

In order to explore the effect of miR-770-5p in HG-treated podocytes, we synthesized the overexpression and knockdown of miR-770-5p. As shown in Figure 2A, the expression level of miR-770-5p was upregulated in HG-treated podocytes transfected with miR-770-5p mimic, and miR-770-5p expression level was downregulated in HG-treated podocytes transfected with anti-miR-770-5p compared with respective control groups. Overexpression of miR-770-5p hindered HG-blocked proliferation, and knockdown of miR-770-5p elevated HG-induced proliferation in podocytes (Figure 2B). As presented in Figure 2C and D, upregulation of miR-770-5p increased HG-triggered apoptosis, and downregulation of miR-770-5p repressed HG-triggered apoptosis of podocytes. Furthermore, HG improved Bax protein level and reduced Bcl-2 and Bad protein levels, while the reintroduction of miR-770-5p relieved these effects. Interestingly, deficiency of miR-770-5p had the opposite results (Figure 2E and F). Taken together, these results indicated that miR-770-5p could reverse HG-triggered proliferation and apoptosis in podocytes.

### E2F3 induced proliferation and suppressed apoptosis of HG-treated podocytes *in vitro*

**Figure 3:**
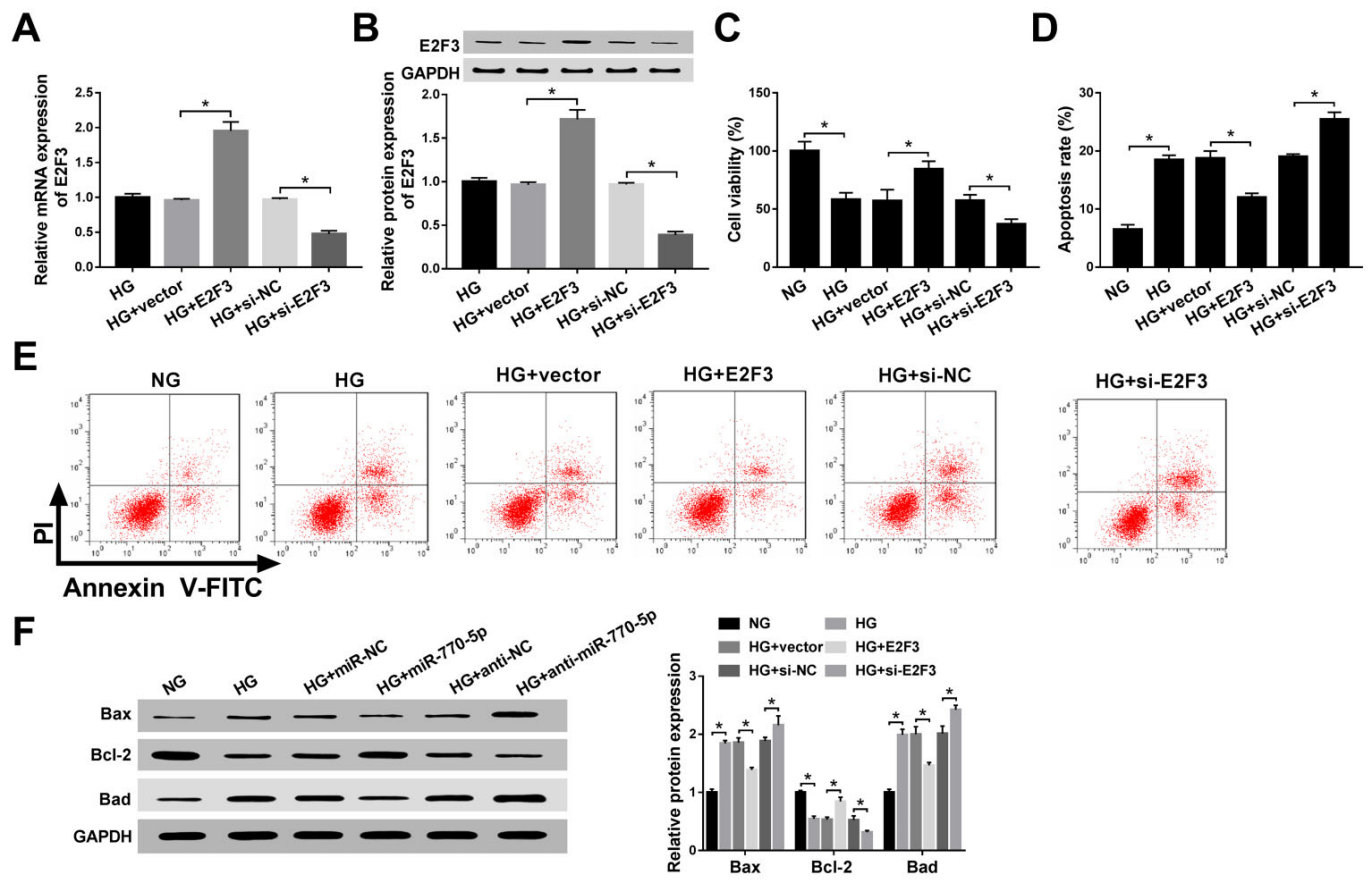
E2F3 promoted proliferation and inhibited apoptosis of high glucose (HG)-treated podocytes *in vitro*. **A** and **B**, Transfection efficiency of E2F3 or si-E2F3 in HG-treated podocytes. **C**, Cell viability in treated podocytes was detected by CCK-8 assay. **D** and **E**, Cell apoptosis rate in treated podocytes was measured by flow cytometry assay. **F**, The expression levels of Bax, Bcl-2, and Bad in treated podocytes were assessed by western blot assay. Data are reported as means±SD. *P<0.05 (ANOVA). NG: normal glucose (5 mM); HG: high glucose (30 mM); NC: negative control.

As mentioned above, E2F3 was downregulated in HG-treated podocytes. Therefore, we over-expressed and knocked down E2F3 in HG-treated podocytes, and transfection efficiency is reported in Figure 3A and B. As illustrated in Figure 3C, the overexpression of E2F3 enhanced, while depletion of E2F3 decreased the proliferative ability of HG-treated podocytes. Moreover, flow cytometry assay showed that the apoptosis rate was rescued because of the upregulation of E2F3 in HG-treated podocytes, whereas its knockdown produced contrary results (Figure 3D and E). E2F3 overexpression led to a decrease of Bax and Bad protein expression and an increase of Bcl-2 in HG-treated podocytes, however, deletion of E2F3 had the opposite effects (Figure 3F). In summary, E2F3 contributed to the proliferation and retarded apoptosis of HG-treated podocytes.

### E2F3 was a target gene of miR-770-5p

**Figure 4:**
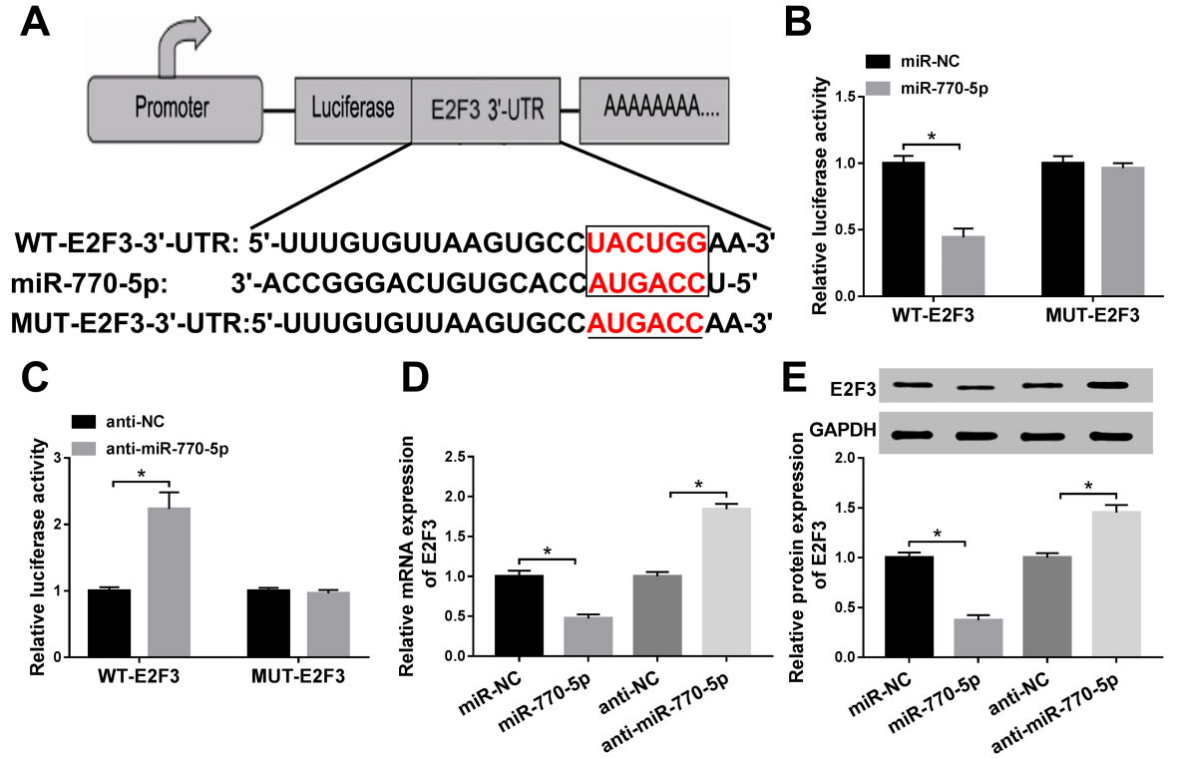
A, Predicted binding sites between miR-770-5p and E2F3 and the sequences of MUT-E2F3. **B** and **C**, Effects of miR-770-5p overexpression or knockdown on luciferase activity of WT-E2F3 and MUT-E2F3 reporters were assessed by dual-luciferase reporter assay in high glucose (HG)-treated podocytes. **D**, Relative E2F3 mRNA level was detected by RT-qPCR HG-treated podocytes transfected with miR-NC, miR-770-5p, anti-NC, and anti-miR-770-5p. **E**, Relative E2F3 protein level was measured by western blot assay in transfected podocytes. Data are reported as means±SD. *P<0.05 (ANOVA and *t*-test). NC: negative control; WT: wild type; MUT: mutated.

Based on the results above, we inferred that miR-770-5p might exert its function by interacting with E2F3. Bioinformatics software TargetScan showed that there was an underlying binding between miR-770-5p and E2F3 (Figure 4A). Dual-luciferase reporter assay further demonstrated the potential relationship in HG-treated podocytes. As presented in Figure 4B and C, miR-770-5p restrained the luciferase activity of WT-E2F3 reporter, and silencing of miR-770-5p reinforced the luciferase activity of WT-E2F3 reporter. Interestingly, overexpression or downregulation of miR-770-5p had little effect on MUT-E2F3 luciferase activities. Besides, miR-770-5p mRNA level was inversely related to E2F3 mRNA level in HG-treated podocytes (Figure 4D). Western blot assay also proved that high expression of miR-770-5p hindered the protein level of E2F3 in HG-treated podocytes, and that lower expression of miR-770-5p elevated E2F3 protein level (Figure 4E). Hence, miR-770-5p could interact with E2F3 to block its expression.

### E2F3 partly abolished the effect of miR-770-5p on HG-triggered proliferation and apoptosis of podocytes

**Figure 5:**
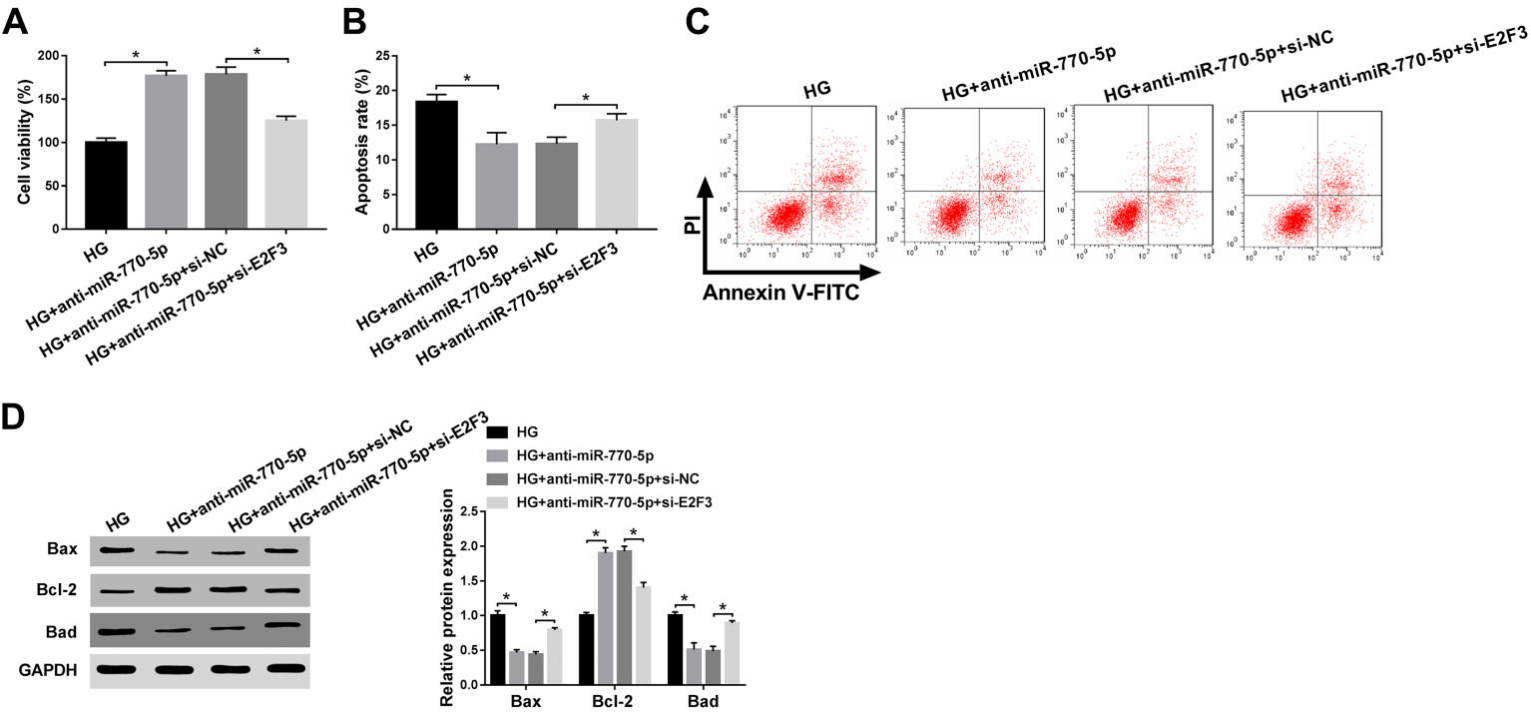
**A**, CCK-8 assay was carried out to detect the impact of miR-770-5p or E2F3 on high glucose (HG)-triggered podocyte viability.**B** and **C**, Flow cytometry analysis was carried out to investigate the influence of miR-770-5p or E2F3 on HG-triggered podocyte apoptosis. **D**, Western blot assay was conducted to detect the expression levels of Bax, Bcl-2, and Bad in treated podocytes. Data are reported as means±SD. *P<0.05 (ANOVA). HG: high glucose (30 mM); NC: negative control.

In addition, to confirm the function of miR-770-5p/E2F3 axis in proliferation and apoptosis of HG-treated podocytes, we implemented the remedial experiment by transfecting HG-treated podocytes with anti-miR-770-5p, anti-miR-770-5p+si-NC (negative control), and anti-miR-770-5p+si-E2F3. As illustrated in Figure 5A, downregulation of miR-770-5p boosted HG-inhibited cell viability, which was effectively weakened by knockdown of E2F3. Flow cytometry assay confirmed that knockdown of miR-770-5p repressed HG-triggered apoptotic rate, while introduction of si-E2F3 abrogated the effect (Figure 5B and C). To further verify the miR-770-5p/E2F3 axis on the apoptosis of HG-treated podocytes, the protein levels of Bax, Bcl-2, and Bad were measured in treated podocytes. The results showed that transfection of anti-miR-770-5p inhibited HG-induced Bax or Bad level and facilitated HG-impeded Bcl-2 level, which was remarkably counteracted by the suppression of E2F3 (Figure 5D). Overall, these data indicated that miR-770-5p-elicited proliferation and apoptosis were reversed by targeting E2F3 in HG-treated podocytes.

### miR-770-5p deficiency blocked HG-stimulated APAF1/caspase9 pathway via downregulating E2F3 in podocytes

**Figure 6:**
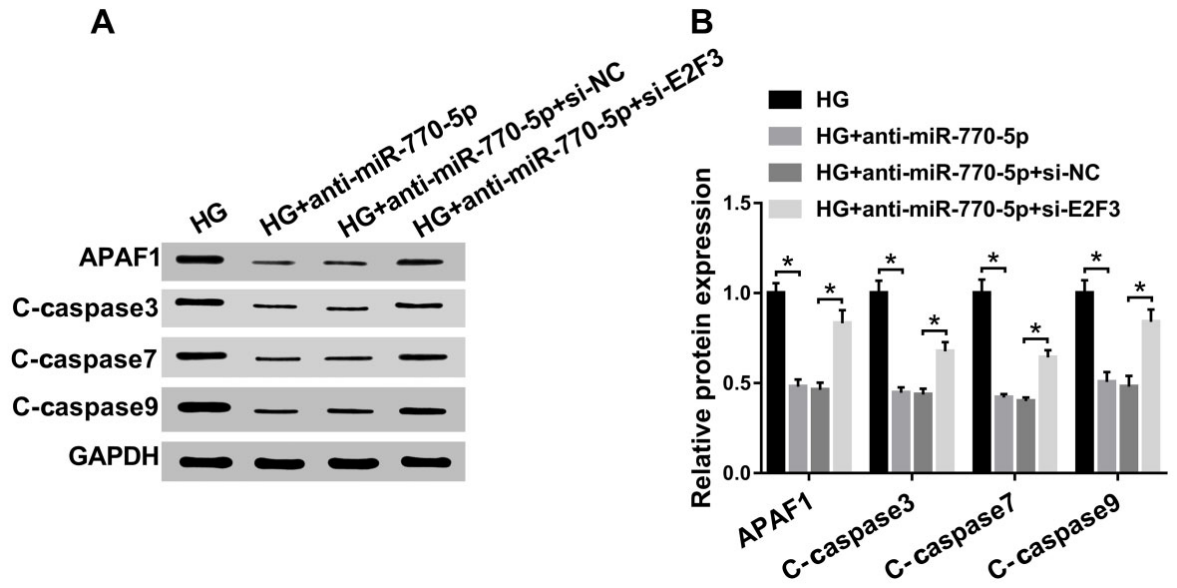
Aand **B**, Protein levels of APAF1, C-caspase3, C-caspase7, and C-caspase9 were detected by western blot assay in high glucose (HG)-treated podocytes transfected with anti-miR-770-5p, anti-miR-770-5p+si-NC, and anti-miR-770-5p+si-E2F3. Data are reported as means±SD. *P<0.05 (ANOVA). HG: high glucose (30 mM); NC: negative control.

Analysis of the APAF1/caspase9 pathway was used to further investigate the influence of miR-770-5p/E2F3 axis on apoptosis in podocytes. miR-770-5p deficiency resulted in a decrease of APAF1, C-caspase3, C-caspase7, and C-caspase9 protein expression, and this suppressive effect was eliminated following knockdown of E2F3 in HG-treated podocytes (Figure 6A and B). Collectively, these results proved that E2F3 partly abrogated the effect of miR-770-5p on HG-stimulated APAF1/caspase9 pathway in podocytes.

## Discussion

In recent years, accumulative evidence has demonstrated that miRNAs are implicated in the development of various kinds of diseases, particularly DN (12,22,23). For instance, Zhou et al. (24) reported that miR-27a elevated migration, invasion, and apoptosis of podocytes by activating PPARγ/β-catenin signaling. Chen et al. (25) revealed that downregulation of miR-21 blocked inflammation and podocyte apoptosis by targeting TIMP3. In addition, miR-770-5p has been proven to be upregulated in HG-treated podocytes and downregulation of miR-770-5p might work as a protective effect in DN (15). A study also showed that E2F3 was downregulated in HG-treated podocytes (18).

In the present study, we investigated the expression miR-770-5p and E2F3 in the treatment of podocytes. RT-qPCR results showed that miR-770-5p was significantly upregulated and E2F3 was significantly downregulated in HG-treated podocytes compared with NG-treated podocytes. Similarly, E2F3 protein level was downregulated in HG-treated podocytes, compared with NG-treated podocytes. The above data indicated the potential involvement of miR-770-5p and E2F3 in DN progression.

Functional analyses suggested that miR-770-5p inhibited proliferation and accelerated apoptosis in HG-induced podocytes. Inversely, downregulation of miR-770-5p expedited proliferation and restrained apoptosis in HG-treated podocytes. Notably, HG-induced apoptosis of podocytes was pointed out to be a typical early characteristic of DN (26). Therefore, knockdown of miR-770-5p might act as a suppressive effect in DN.

Additionally, the impact of E2F3 on proliferation and apoptosis in HG-treated podocytes were further probed. The results showed that E2F3 led to a notable enhancement in cell viability and a decrease in apoptotic rate in HG-treated podocytes. On the contrary, siRNA-mediated E2F3 silencing elicited a decline in cell viability and an increase in apoptotic rate in HG-treated podocytes, implying that E2F3 deletion might work as a promoter in DN. Currently, a growing body of research identified that miRNA exerted its function by interacting with mRNAs (27,28). Thus, E2F3 as the target of miR-770-5p in HG-treated podocytes was predicted by TargetScan and further verified by dual-luciferase reporter assay. Moreover, in the current study, we also observed that miR-770-5p expression level was negatively correlated with E2F3 expression level in HG-treated podocytes. Therefore, to further explore whether the biological function of miR-770-5p in HG-treated podocytes was mediated by targeting E2F3, we conducted rescue experiments. The results confirmed that miR-770-5p deficiency boosted proliferation and repressed apoptosis in HG-treated podocytes, while reintroduction of si-E2F3 partially reversed these effects. In other words, miR-770-5p silencing suppressed podocyte injury partly by targeting E2F3.

A previous study indicated that E2F3 regulates the expression of APAF1, which is necessary for E2F3-induced apoptosis (29). APAF1, an essential stimulator of caspase activity, could form apoptotic complexes with cytochromes and caspase9 (30,31). Thus, we investigated whether miR-770-5p was involved in the effect of APAF1/caspase9 pathway on podocyte apoptosis. Western blot results showed that miR-770-5p downregulation hindered HG-induced improvement of APAF1, C-caspase3, C-caspase7, and C-caspase9, which was significantly eliminated by si-E2F3. Additionally, we will continue to explore *in vivo* experiments of miR-770-5p and E2F3 on podocytes, as well as other biological activities, such as migration and invasion.

In conclusion, our findings revealed that miR-770-5p might contribute to HG-triggered podocyte apoptosis through targeting E2F3, providing a useful approach for deciphering the mechanisms used by miRNAs in the pathogenesis of DN.
